# Clinical use of polygenic scores in type 2 diabetes: challenges and possibilities

**DOI:** 10.1007/s00125-025-06419-1

**Published:** 2025-04-05

**Authors:** Rashmi B. Prasad, Liisa Hakaste, Tiinamaija Tuomi

**Affiliations:** 1 Lund University Diabetes Centre, Department of Clinical Sciences, Genetics and Diabetes, CRC, Lund University, Malmö, Sweden; 2 Institute for Molecular Medicine Finland (FIMM), Helsinki University, Helsinki, Finland; 3 Folkhalsan Research Centre, Helsinki, Finland; 4 Helsinki University Hospital, Abdominal Centre/Endocrinology, Helsinki, Finland

**Keywords:** Ancestries, Comorbidities, Genetic risk, Mechanisms, Polygenic scores, Prediction, Review, Screening, Subtypes, Type 2 diabetes

## Abstract

Resulting from a combination of genetic and environmental factors, type 2 diabetes is highly heterogeneous in manifestation and disease progression, with the only common feature being chronic hyperglycaemia. In spite of vigorous efforts to elucidate the pathogenetic origins and natural course of the disease, there is still a lack of biomarkers and tools for prevention, disease stratification and treatment. Genome-wide association studies have reported over 1200 variants associated with type 2 diabetes, and the decreased cost of generating genetic data has facilitated the development of polygenic scores for estimating an individual’s genetic disease risk based on combining effects from most—or all—genetic variants. In this review, we summarise the current knowledge on type 2 diabetes-related polygenic scores in different ancestries and outline their possible clinical role. We explore the potential applicability of type 2 diabetes polygenic scores to quantify genetic liability for prediction, screening and risk stratification. Given that most genetic risk loci are determined from populations of European origin while other ancestries are under-represented, we also discuss the challenges around their global applicability. To date, the potential for clinical utility of polygenic scores for type 2 diabetes is limited, with such scores outperformed by clinical measures. In the future, rather than predicting risk of type 2 diabetes, the value of polygenic scores may be in stratification of the severity of disease (risk for comorbidities) and treatment response, in addition to aiding in dissecting the pathophysiological mechanisms involved.

**Graphical Abstract:**

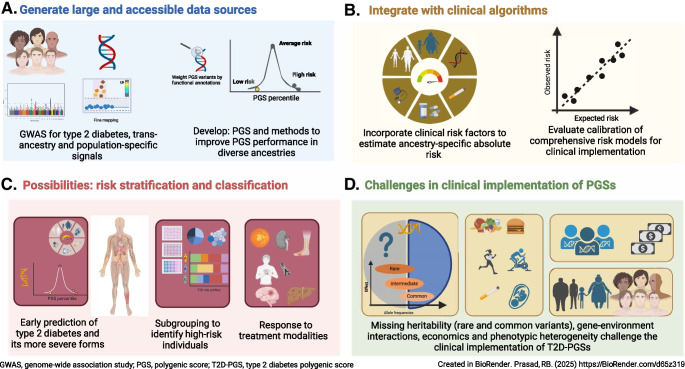

**Supplementary Information:**

The online version contains a slideset of the figures for download available at 10.1007/s00125-025-06419-1.

## Introduction

**Fig. 1:**
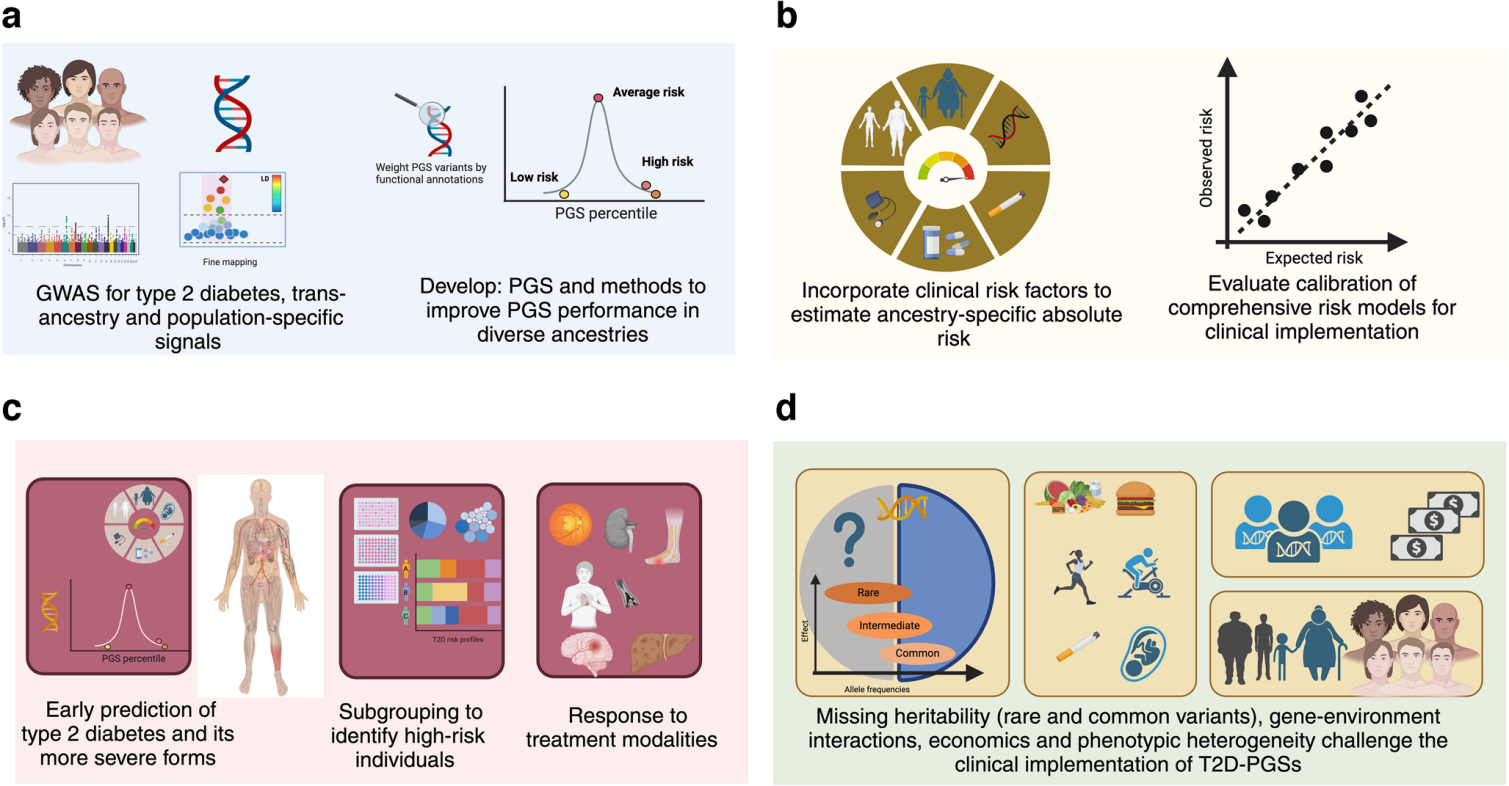
Clinical utility of polygenic scores: possibilities and challenges. (**a**) Creation of optimal polygenic scores for type 2 diabetes requires generation of large, accessible data sources covering diverse ancestries to identify transancestry as well as population-specific signals. Simultaneous method development to improve PGS performance in diverse ancestries will also be vital to facilitate opportunities for clinical application. (**b**) Integration of PGSs with clinical risk factors such as age, body composition and exposures (e.g. smoking) and evaluating the calibration of risk models will be important steps towards clinical implementation. (**c**) PGSs have potential for use in early prediction of type 2 diabetes and its more severe forms, subgrouping and evaluating response to treatment modalities. (**d**) Several challenges such as missing heritability (pertaining to both rare and common variants), gene–environment interactions, economics and phenotypic heterogeneity currently challenge the clinical implementation of T2D-PGSs. GWAS, genome-wide association study; T2D, type 2 diabetes. Created in BioRender. Prasad, R. (2025) https://BioRender.com/g39f279. This figure is available as part of a downloadable slideset

**Fig. 2:**
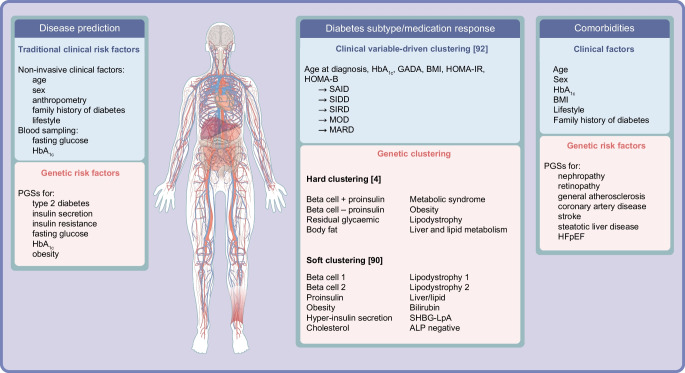
Type 2 diabetes: risk stratification and classification. Current prediction and screening methods for type 2 diabetes, diabetes subtypes/medication response and comorbidities are based on traditional clinical risk factors. Adding PGSs to current practices as one risk factor among others may aid in the detection of individuals at risk of type 2 diabetes and associated complications at early stages of disease development and allow for better subclassification of disease. Examples of PGS models assessed for prediction of susceptibility to disease and comorbidites include those for risk factors such as obesity and coronary artery disease. PGSs also provide clues to the underlying pathophysiology of diabetes subtypes defined by both clinical variable-derived and genetic clustering. Ahlqvist et al [92] defined five clinical variable-derived clusters. Suzuki et al [4] reported eight genetic clusters using a hard clustering approach, allowing for one SNP to belong to only one cluster. Smith et al [90] identified 12 clusters using a soft clustering approach whereby a SNP can associate with more than one cluster. ALP, alkaline phosphatase; HFpEF, heart failure with preserved ejection fraction; LpA, lipoprotein A; MARD, mild age-related diabetes; MOD, mild obesity-related diabetes; SAID, severe autoimmune diabetes; SHBG, sex hormone-binding globulin; SIDD, severe insulin-deficient diabetes; SIRD, severe insulin-resistant diabetes. This figure is available as part of a downloadable slideset

Type 2 diabetes is one of the most common diseases in the world, with diagnosis involving only chronic hyperglycaemia and exclusion of other specific causes [1]. It manifests as heterogeneous combinations of varying degrees and mechanisms of insulin deficiency and insulin resistance together with varying rates and types of comorbidities. Lifestyle factors have a significant impact on diabetes risk and progression but there is also a major genetic component [2, 3]. While recent breakthroughs have identified over 1200 common and rare associated genetic variants [4, 5], several of which are associated with related metabolic traits [5–9], the low effect sizes of these variants mean that they have little clinical utility as predictive or diagnostic markers at individual level. However, combining the effects of several variants as polygenic scores (PGS) (also known as genetic risk scores or polygenic risk scores) may be useful. In the review, we discuss the clinical translation potential of type 2 diabetes PGSs (T2D-PGSs) and related PGSs for (1) risk prediction and screening of type 2 diabetes and its comorbidities and (2) risk stratification in clinical care, paying special attention to usability in populations from different ancestries. An outline of the review is presented in Fig. 1. An overview of type 2 diabetes risk stratification and classification is presented in Fig. 2.

## PGSs for prediction and screening of type 2 diabetes

Numerous risk calculators have been developed for the early detection of type 2 diabetes [10–12], as it can remain asymptomatic for years despite the development of comorbidities [10, 13]. Typically, risk calculators are based on traditional risk factors such as age, sex, ethnicity, history of hypertension, BMI, waist circumference and family history of diabetes (Fig. 2). Some risk calculators also consider fasting plasma glucose and HbA_1c_. Usually, screening is considered in adults of any age with overweight or obesity and in all individuals aged ≥45 years, as BMI is the strongest risk factor for type 2 diabetes [14]. Performance metrics used for prediction, screening and diagnostic models are summarised in the text box [15, 16].


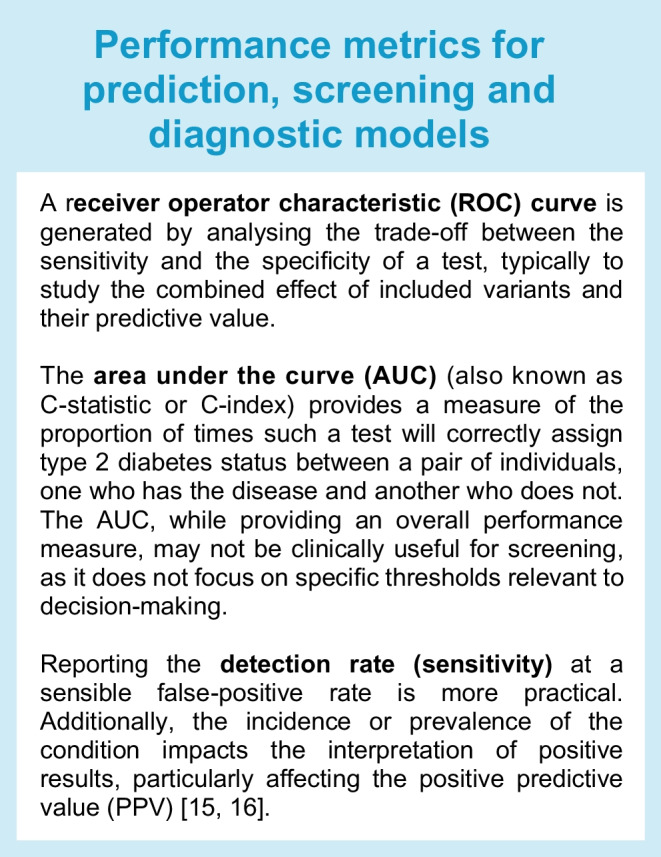


The high degree of type 2 diabetes heritability (69%) among European populations aged 35–60 years [17] has led to expectations that adding T2D-PGSs to models would considerably improve type 2 diabetes risk discrimination. However, the results of such studies have been modest. The largest T2D-PGS to date is a metaPGS based on summary statistics from 44 genome-wide association studies (GWAS) performed in both European and non-European genetic ancestries for type 2 diabetes and its risk factors. Compared with the QDiabetes risk prediction score [12] comprising traditional risk factors, the metaPGS had a larger C-index for 10 year type 2 diabetes risk prediction (0.716; 95% CI 0.708, 0.723) than all individual traditional risk factors, including family history (C-index: 0.687; 95% CI 0.679, 0.695), except for BMI (C-index: 0.780; 95% CI 0.773, 0.787) and HbA_1c_ (C-index: 0.826; 95% CI 0.819, 0.833) [18]. In addition, when incorporating the metaPGS into absolute risk predictions made by QDiabetes risk scores, the metaPGS significantly improved the risk stratification of QDiabetes, increasing the number of correctly classified future incident type 2 diabetes cases.

In some situations, such as in younger individuals and individuals without obesity, T2D-PGSs have clear advantages. While the performance of the clinical models varies with age and adiposity [10], the genetic variants remain constant [19], allowing for risk prediction either at an early age before the typical risk factors manifest, or in normal-weight individuals. It is especially important to identify individuals with younger-onset type 2 diabetes, as they are prone to more severe forms of disease and higher mortality rates [20, 21].

Among many populations, especially in East and South-East Asia, a considerable proportion (up to 60%) of people with type 2 diabetes are of normal weight or lean. Even in Western countries, up to 25% of individuals with type 2 diabetes have a normal BMI [22]. Overall, T2D-PGSs have revealed differences in both the relative and the absolute risk of type 2 diabetes among individuals in all categories of BMI, with the risk of incident type 2 diabetes being considerably higher in individuals with the highest T2D-PGS in all BMI categories in both men and women [23, 24]. The genetically determined risk appears to be higher for individuals at the lower end of each BMI range than for those at the higher end (Tables 1, 2, 3 and 4) [23–25], pointing to a stronger genetic risk or specific pathophysiology in those without the classical type 2 diabetes phenotype. In a Chinese population, individuals with normal weight showed higher partitioned polygenic scores (pPSs; see text box: Types of polygenic scores) for beta cell dysfunction and lipodystrophy than those with overweight, while the pPSs for obesity were associated with faster progression to clinical requirement of insulin treatment [26]. Furthermore, in a separate study of a US population, each SD increase in a T2D-PGS was associated with a decrease in age at diagnosis of type 2 diabetes by 1.3 years. In addition, in individuals without type 2 diabetes, an elevated T2D-PGS increased the odds of reported high blood sugar by 23% and type 2 diabetes by 43% within a year [27].


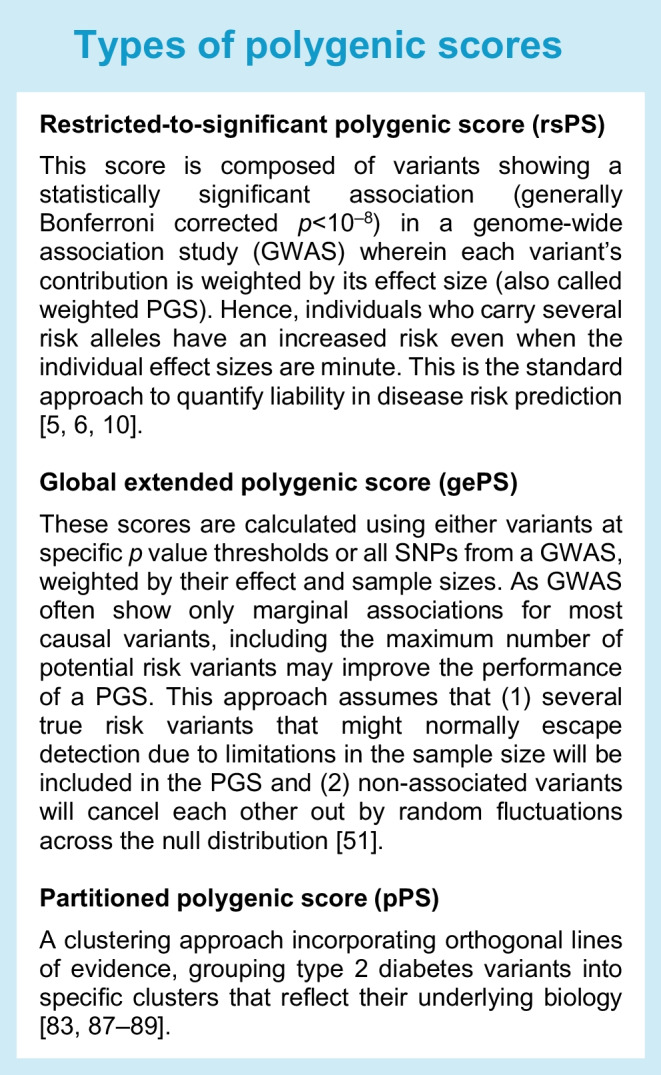


**Table 1:** Heritability potential of various strata of risk factors assessed using heritability h2 (LDSC regression) [24]

Study population	Groups/model	Heritability h2^a^ (LDSC regression)
BBJ *n*(T2D)=27,642, *n*(control)=70,242	Unstratified	0.145
UKBB *n*(T2D)=27,642, *n*(control)=70,242		0.131
BBJ *n*(T2D)=13,821, *n*(control)=35,121	BMI stratified, two groups	+84.5% in lowest vs highest BMI group (0.198 vs 0.107)
UKBB *n*(T2D)=13,821, *n*(control)=35,121	BMI stratified, two groups	+64.7% in lowest vs highest BMI group (0.152 vs 0.092)
BBJ *n*(T2D)=9214, *n*(control)=23,414	BMI stratified, three groups	Non-significant difference between lowest and highest BMI groups, but a similar trend as in the BMI-stratified datasets separated into two groups
UKBB *n*(T2D)=9214, *n*(control)=23,414	BMI stratified, three groups	Non-significant difference between lowest and highest BMI groups, but a similar trend as in the BMI-stratified datasets separated into two groups

^a^h2 is broad-sense heritability that describes the contribution of genetic variation to the variation in a given trait

BBJ, BioBank Japan; LDSC, linkage disequilibrium score; T2D, type 2 diabetes; UKBB, UK Biobank

**Table 2:** Prediction potential of various strata of risk factors assessed using liability *R*^2^ [24]

Study population	Groups/model	Liability *R*^2^ (pseudo-*R*^2^ value)
BBJ *n*(T2D)=27,642, *n*(control)=70,242	Unstratified	0.072
UKBB *n*(T2D)=27,642, *n*(control)=70,242	Unstratified	0.051
Lowest BMI group vs unstratified		
BBJ *n*(T2D)=13,821, *n*(control)=35,121	BMI stratified, two groups	+22% in lowest BMI group vs unstratified (0.088 vs 0.072)
UKBB *n*(T2D)=13,821, *n*(control)=35,121	BMI stratified, two groups	+23.5% in lowest BMI group vs unstratified (0.063 vs 0.051)
BBJ *n*(T2D)=9214, *n*(control)=23,414	BMI stratified, three groups	+30.6% in lowest BMI group vs unstratified (0.094 vs 0.072)
UKBB *n*(T2D)=9214, *n*(control)=23,414	BMI stratified, three groups	+21.6% in lowest BMI group vs unstratified (0.062 vs 0.051)
Lowest vs highest BMI groups		
BBJ *n*(T2D)=13,821, *n*(control)=35,121	BMI stratified, two groups	+60% in lowest vs highest BMI group (0.088 vs 0.055)
UKBB *n*(T2D)=13,821, *n*(control)=35,121	BMI stratified, two groups	+57.5% in lowest vs highest BMI group (0.063 vs 0.040)
BBJ *n*(T2D)=9214, *n*(control)=23,414	BMI stratified, three groups	+100% in lowest vs highest BMI group (0.094 vs 0.047)
UKBB *n*(T2D)=9214, *n*(control)=23,414	BMI stratified, three groups	+72.2% in lowest vs highest BMI group (0.062 vs 0.036)

BBJ, BioBank Japan; T2D, type 2 diabetes; UKBB, UK Biobank

**Table 3:** Prediction potential of various strata of risk factors assessed using AUC [25]

Study population	Groups/model	AUC (95% CI)
UKBB *n*(total)=121,113 (2544 incident T2D)	GRIT-T2D: age, sex, T2D-PGS, antihypertensives, statin, CVD, FH, smoking status, gestational diabetes	0.810 (0.803, 0.818)
	GRIT-T2D+: GRIT-T2D + TG, HDL, systolic BP	0.827 (0.820, 0.834)
Stratified by age: <55 vs ≥55 years		
UKBB *n*(total)=121,113 (2544 incident T2D)	GRIT-T2D age <55 years	0.846 (0.830, 0.861)
	GRIT-T2D age ≥55 years	0.780 (0.770, 0.789)
	GRIT-T2D+ age <55 years	0.862 (0.847, 0.877)
	GRIT-T2D+ age ≥55 years	0.798 (0.789, 0.807)
Stratified by BMI: <30 vs ≥30 kg/m^2^		
UKBB *n*(total)=121,113 (2544 incident T2D)	GRIT-T2D BMI <30 kg/m^2^	0.786 (0.773, 0.799)
	GRIT-T2D BMI ≥30 kg/m^2^	0.708 (0.695, 0.721)
	GRIT-T2D+ BMI <30 kg/m^2^	0.808 (0.795, 0.820)
	GRIT-T2D+ BMI ≥30 kg/m^2^	0.734 (0.721, 0.746)

FH, family history of diabetes; GRIT-T2D, Genomics-enhanced Risk Tool; TG, triacylglycerol; T2D, type 2 diabetes; UKBB, UK Biobank

**Table 4:** Prediction potential of various strata of risk factors assessed using T2D-PGS HR for incident type 2 diabetes [23]

Study population	Groups/model	T2D-PGS HR (95% CI)^a^
Stratified by BMI		
UKBB *n*(total)=431,658 (17,259 incident T2D)	Normal BMI^b^. Model: age + sex + BMI + FH + genetic array + first four genetic principal components + PGS × BMI	2.21 (1.92, 2.56)
	Overweight^c^. Model: age + sex + BMI + FH + genetic array + first four genetic principal components + PGS × BMI	2.19 (2.04, 2.35)
	Obese^d^. Model: age + sex + BMI + FH + genetic array + first four genetic principal components + PGS × BMI	1.80 (1.70, 1.91)
Stratified by first-degree FH		
UKBB *n*(total)=431,658 (17,259 incident T2D)	First-degree FH. Model: age + sex + BMI + FH + genetic array + first four genetic principal components + PGS × FH	1.81 (1.68, 1.95)
	No first-degree FH^b^. Model: age + sex + BMI + FH + genetic array + first four genetic principal components + PGS × FH	2.06 (1.96, 2.17)

^a^Cox proportional hazards model; highest quintile vs middle PGS quintiles

^b^BMI ≥18.5 to <25 kg/m^2^

^c^BMI ≥25 to <30 kg/m^2^

^d^BMI ≥30 kg/m^2^

*p*<0.001 for both comparisons (BMI strata and first-degree FH strata)

FH, family history of diabetes; T2D, type 2 diabetes; UKBB, UK Biobank

It has been suggested that family history of diabetes should be used for prediction of type 2 diabetes instead of T2D-PGSs. However, family history and PGSs are independent and not interchangeable measures. A PGS provides complementary information on inherited disease susceptibility [28], especially for those without a known first-degree family history of diabetes (Tables 1, 2, 3 and 4) [23]. Moreover, not all people know their family history, and lack of a family history of diabetes may also be a consequence of the healthier lifestyle of parents protecting against diabetes in their offspring, despite them having a high genetic risk that may have been passed to their offspring. Of note, individuals with a high risk of diabetes often also have a high risk of CVD, which may lead to premature death before the diagnosis of diabetes. Another point has been that knowledge of one’s own genetic risk can cause anxiety. This can be counteracted by information. Obviously, individuals with a high genetic risk are not destined to develop type 2 diabetes, and protective lifestyle changes have been shown to attenuate the risk of diabetes in those with a high T2D-PGS [29, 30]. On the other hand, as a high T2D-PGS itself has been associated with unhealthy dietary and physical activity habits [31], genetic information may encourage the adoption of a healthier lifestyle. Indeed, receiving personal risk data for CVD (including polygenic risk) motivated positive health behaviour changes and healthcare contacts among a cohort of over 7000 individuals with a mean age of 56 years, which supports the integration of genomic information into clinical risk calculations [32].

## PGSs for prediction and screening of comorbidities

As the individual and financial burdens of type 2 diabetes largely derive from symptoms, screening and treatment of vascular comorbidities (nephropathy, retinopathy, neuropathy, coronary and peripheral artery disease, cerebrovascular disease), it is of utmost importance to identify individuals at high risk of these comorbidities [33]. Risk calculators based on clinical factors have suboptimal predictive power [34–36], and similar screening practices are usually applied for all individuals. The predictions rely heavily on the presence of hyperglycaemia, which is a strong predisposing factor [37], but optimal blood glucose levels do not guarantee protection against comorbidities. Also, screening for diabetic nephropathy relies on existing signs of kidney damage: albuminuria or a decline in GFR [38]. PGS for nephropathy may help stratify risk at earlier stages as suggested in a recent systematic review [39].

T2D-PGSs as such are associated with increased risks of retinopathy, kidney disease, peripheral artery disease, neuropathy and coronary artery disease (CAD), pointing to genomic pathways that link type 2 diabetes to vascular outcomes [7], but there are few studies on PGSs designed for comorbidities of diabetes (Fig. 2). A study among 6079 individuals with type 2 diabetes of European, Hispanic, African and other ancestries showed that individuals in the top PGS decile for retinopathy had retinopathy 1.8 times more often and earlier than those in the bottom decile [40]. PGSs for type 2 diabetes and CAD independently predict future cardiovascular mortality risk [41], and a large biobank study indicated that PGSs for CAD had potential for clinical utility, at least for those of European ancestry [42]. However, the use of PGSs as an addition to clinical risk models has also been challenged with regard to CAD, stroke and heart failure [43]. Given the differences in clinical presentation of CAD and stroke in people with and without diabetes (the former having more extensive disease also affecting the small arteries), it can be speculated that a general CAD-PGS might perform less well than a T2D-specific CAD-PGS in individuals with type 2 diabetes. However, at least among individuals of European and South Asian descent, no evidence of different genetic architecture of CAD was noted between those with type 2 diabetes and those without [44].

## PGSs for predicting response to glucose-lowering medications

It would be clinically useful to employ PGSs to predict individual responses to glucose-lowering medications; however, data in this area are limited [45]. To date, instead of using partitioned scores, studies have used only known loci for type 2 diabetes and related traits. Of note, in addition to glycaemic response, it would also be important to look for any associations with side effects.

Metformin is the most common initial treatment for type 2 diabetes; however, many individuals do not achieve adequate glycaemic control on metformin, resulting in delays in commencement of other therapies. It has been suggested that the metformin response in type 2 diabetes is associated with variants in, for example, *SLC22A1*, *ATM* and *SLC2A2;* however, the replicability of the *SLC22A1* locus associations has been inconsistent, potentially owing to differences between studies in study design, population characteristics or disease stage [46–50]. Interestingly, individuals with a high global extended polygenic score [51] (gePS; see text box: Types of polygenic scores) for fasting glucose had a reduced glucose response to metformin in the Study to Understand the Genetics of the Acute Response to Metformin and Glipizide in Humans (SUGAR-MGH) [52], suggesting that PGSs may aid in choosing the primary treatment for type 2 diabetes.

Response to sulfonylureas may also be heritable. Individuals with a high restricted-to-significant polygenic score (rsPS; see text box: Types of polygenic scores) for type 2 diabetes had greater acute and sustained responses to sulfonylureas in the SUGAR-MGH study, and many variants (e.g. in *CYP2C9*, *KCNJ11*, *TCF7L2*, *GXYLT1* and *SLCO1B1*) were also independently associated with this trait [53]. Other studies have reported the association of *GLP1R* and *ARBB1* variants with response to glucagon-like peptide-1 receptor agonists [54], *GLP1R* and *DPP4* variants with response to dipeptidyl peptidase-4 inhibitors [55, 56], and *SLC5A2* variants with response to sodium–glucose cotransporter 2 inhibitors [57].

T2D-PGSs have also been associated with insulin treatment (as a proxy for advanced disease) in diverse populations [27, 58]. Among a South Asian population, population-specific pharmacogenetic variant profiles were marked by an excess of alleles associated with poor treatment response to various non-insulin glucose-lowering drug classes. This calls for further pharmacogenetic studies in multiple ancestries and reconsideration of dosage recommendations for glucose-lowering medications to ensure optimal efficacy and safety [59].

## PGSs for type 2 diabetes in different ancestries

As GWAS findings have largely been based on populations of European ancestry, it is unsurprising that PGSs often fail to predict disease risk in other populations. The genetic architecture of populations varies markedly (e.g. effect sizes, allele frequencies and patterns of linkage disequilibrium), as do the genetic associations with diseases [60]. In recent years, there has been a rapid increase in the inclusion of participants from diverse ancestral backgrounds, and several type 2 diabetes GWAS have been performed on non-European populations [9, 61, 62]. Indeed, the latest GWAS of ~2.5 million participants (17% with type 2 diabetes) included 40% of individuals with non-European ancestry and identified 1289 independent signals, of which 46% were attributed to inclusion of previously under-represented ancestry groups [4]. While the majority of the discovered loci have similar effects across ancestries, ancestry-specific loci have also been identified [5], such as loci in *SLC16A11* and *SLC16A13* in Latin American individuals [63], *TMEM163* in Asian Indian individuals [64], *DNER* in American Indian individuals [65], *SCTR*, *GP2* and *ZNF257* in Japanese individuals [66], *ZRANB3*, *AGMO*, *ANKH*, *INS-IGF2-KCNQ1, TGFB1* and *AGMO* in African individuals [4, 5, 67–69], and *UBE2E2*, *PAX4*, *KLF14*, *ANK1* and *INS* in East Asian individuals [4]. Adding ancestry-specific loci to European T2D-PGSs outperformed the European T2D-PGSs alone in different populations [62, 70, 71]. For instance, adding ancestry-specific loci to European T2D-PGSs boosted type 2 diabetes prediction in a population from continental Africa [71–73], and ancestry-specific PGSs outperformed European-based PGSs in South Asian populations, with a more than 20-fold higher type 2 diabetes risk predictability among individuals in the top (ninth) compared with the middle (fifth) decile, showing the sensitivity and effectiveness of the PGS models even at the lower extremes of the distribution [74]. In another study, T2D-PGS based on South Asian individuals showed an approximately fourfold higher risk between the top and the bottom quartiles [75]. Polfus et al showed that, in large sample sizes and using multiancestry weights, both of which are more likely to accurately reflect the true causal effect of a variant, a multiancestry PGS outperformed a population-specific PGS [67].

Approaches to increase the predictive potential of PGSs could include adding rare ancestry-specific variants to the common variant-based PGSs and considering that associations may indicate mechanistic pleiotropy. For example, in a GWAS on HbA_1c_ levels in European, East Asian and South Asian populations, some variants predicted type 2 diabetes risk whereas others influencing HbA_1c_ through erythrocytic pathways did not [76]. A dominant effect of the erythrocytic *G6PD* variant on type 2 diabetes was seen only in individuals with African American ancestry [76]. These approaches were combined to construct PGSs for HbA_1c_ based on a selection of rare and common variants, and including variants in genes with known erythrocytic roles. Including 22 common and 21,293 rare (across 154 genes; 73% observed in fewer than three people) variants augmented the diagnostic potential compared with PGSs including only common variants [77]; adding other dimensions such as metabolite data further enhanced type 2 diabetes risk prediction [78].

A type 2 diabetes predictive model including a PGS, physical measurements and clinical risk factors increased the prediction performance compared with models without the PGS, and predicted conversion from normal glucose tolerance to prediabetes to type 2 diabetes, in 5490 Korean individuals [58]. A polyexposure score (PXS) combining multiple lifestyle and exposure factors showed modest improvement in predicting risk over clinical factors and PGSs in White British individuals from the UK Biobank [73]. Applying a similar PXS in addition to clinical factors and PGSs may also improve prediction in non-European populations; however, the variability in exposures between populations and the gene–environment interactions may add to the complexity in transferring European-derived GWAS scores across ancestries.

## Potential challenges around clinical implementation of PGSs in diverse ancestries

**Fig. 3:**
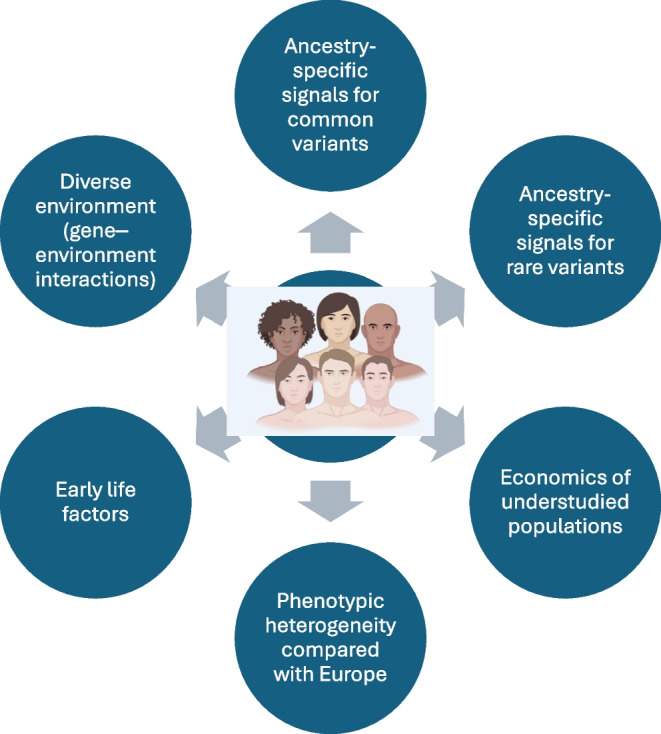
Challenges around clinical implementation of PGSs in understudied/diverse populations. Insufficient knowledge of ancestry-specific signals for common and rare variants can decrease their prediction potential. Inadequate information on early life factors, such as birthweight, maternal nutrition, early growth patterns and childhood obesity, can interact with genetic susceptibility to modulate T2D risk. This can enhance risk stratification and provide information on gene–environment interactions. Vast variations in environmental conditions, including cultural diversity, and economic challenges such as lack of access to infrastructure can also present significant challenges for the clinical implantation of T2D-PGSs. Moreover, the wide heterogeneity in disease manifestation (e.g. lean type 2 diabetes in South Asian populations) and underlying genetic architecture, and differences in phenotypic measures compared with European populations, renders the application of European-derived PGSs especially challenging. This figure is available as part of a downloadable slideset

The clinical implementation of PGSs in diverse ancestries faces several challenges (Fig. 3). While the inclusion of participants from different ancestral backgrounds has increased, and European-derived scores may be applicable to other ancestries, the genetic background of type 2 diabetes in non-European populations is still underexplored. There is some promise in the improving, albeit more complex, methodology for calculating multiple-ancestry PGSs, which leverages machine learning/artificial intelligence (AI) and reported summary statistics [79, 80]. The contribution of known type 2 diabetes genetic signals to heritability in non-European populations remains unclear, and ancestry-specific variants are yet to be identified, complicated by accessibility and economic barriers. Phenotypic differences across ethnicities also affect risk variations. For example, BMI cut-offs for obesity (≥30 kg/m^2^) were developed in European populations but may not be appropriate for other groups; in this regard, Asian populations exhibit higher type 2 diabetes prevalence at lower BMI and younger age, highlighting the need to consider population-specific cut-offs [81]. Moreover, the genetic predisposition captured by PGSs may render an individual’s risk assessment incomplete, as it may not fully account for the influence of early life factors and diverse environments, as well as their interactions with type 2 diabetes genetic loci, complicating PGS development and utility [4, 82].

## Using PGSs to unravel the heterogeneity of type 2 diabetes

Hyperglycaemia may be driven by various mechanisms that directly or indirectly affect insulin secretion and action [83]. Most individuals are likely to have mixed phenotypes arising from the contribution of several pathways, which can be difficult to deduce from clinical measurements [72]. Variants associated with type 2 diabetes may reflect these processes independently or through pleiotropic effects. Different approaches have been used to partition diabetes subtypes using clinical and/or genetic data, but the tentative subtypes are not yet ready to be used in the clinic. More advanced methods using AI on detailed phenotypes together with genetic information may allow for better subclassification and identification of individuals with high risk for the disease and development of complications [62, 84, 85].

### The PGS approach

Using machine learning, known variants have been grouped into ‘clusters’ or pPSs (see text box: Types of polygenic scores), representing different biological pathways (Figs 1 and 2). Each individual receives a score for each cluster, giving a more versatile view of their genetic risk. Applying pPSs to previously reported type 2 diabetes loci, six clusters were initially defined for impaired lipid metabolism, insulin secretion 1 and 2, adiposity, insulin action and insulin secretion/action [86]. Also using ‘hard clustering’, Suzuki et al defined eight clusters in the multiancestry GWAS comprising 40% non-European ancestry participants and allowed individuals to belong to only one cluster (Fig. 2). These clusters were characterised by distinct profiles of cardiometabolic trait associations; the obesity and lipodystrophy clusters associated with CAD, with the former also associating with peripheral artery disease and end-stage diabetic nephropathy (Fig. 2) [4]. Ancestry-related differences were observed: allelic effects were strongest in East Asian individuals for beta cell dysfunction clusters, and in European individuals for insulin resistance clusters. After accounting for BMI variation across ancestry groups, significant differences in association signals persisted between African and European cohorts, but not between African and East Asians cohorts [4]. In addition, the heterogeneity in effects for beta cell dysfunction clusters seemed to be accounted for by BMI differences between East Asian, European and African cohorts [4].

To allow for a particular variant to be associated with several clusters, Udler et al applied ‘soft clustering’ to 95 type 2 diabetes loci (associated with 47 diabetes-related traits) and identified five robust clusters, two related to insulin secretion (‘beta cell’ and ‘proinsulin’) and three to insulin response or action (‘obesity’, ‘lipodystrophy’ and ‘liver/lipid’) [87]. The obesity and lipodystrophy clusters were associated with increased blood pressure and hypertension, and the latter was also associated with CAD. The liver/lipid cluster was associated with decreased CAD and reduced renal function, suggesting a shared pathway [88, 89], perhaps via insulin resistance. Further clustering of 650 type 2 diabetes variants with 110 trait associations identified 12 clusters (recapturing the previously defined five clusters) [90] (Fig. 2). Applying this multiancestry pPS to British South Asian individuals revealed that genetic predisposition to insulin deficiency and lipodystrophy was linked to earlier onset of type 2 diabetes, more rapid progression to complications, insulin dependence and diminished response to medication [91].

An advantage of this approach is the consistent categorisation of genetically driven propensity to malfunction of certain molecular pathways and propensity for related metabolic disease outcomes. However, limitations exist. The assignment of the variants to certain pathways and clusters is based on limited numbers of individuals with deep phenotyping data. As the clusters are defined by allele distributions and scores are continuous, the boundaries can be unclear. Also, while heritability of type 2 diabetes is at most 69% and the known risk variants explain only about 50% of the heritability, these genetic clusters are not comprehensive. Adding more variants may facilitate the identification of more clusters with several possible permutations and combinations, while also allowing for refining for more precise definitions. Population-specific scores and environmental contributions will need to be taken into consideration for clinical applicability.

### The clinical variable-based approach

This approach involves using clinical variables to categorise individuals. Data-driven machine learning performed on commonly available variables (age at diagnosis, sex, BMI, HbA_1c_) and HOMA2-B and HOMA2-IR in newly diagnosed individuals with diabetes from southern Sweden identified five reproducible clusters: severe autoimmune (SAID), insulin-deficient (SIDD) and insulin-resistant (SIRD) diabetes and mild obesity-related (MOD) and age-related (MARD) diabetes (Fig. 2) [92]. The groups (replicated in several populations [81, 93]) differ regarding clinical features, complication risk and disease progression [92]. Despite partially different genetic associations, for example SIRD uniquely associated with a fasting insulin PGS, while most tested PGSs associated with more than one cluster [94], no subgroup-specific PGSs are available. When these subgroups were applied to Indian cohorts, the PGS associations were partially replicated, but some were unique to this population. For instance, association with a liver-related PGS was seen in India but not Europe and was related to poor liver growth in utero and other early life factors that may contribute to the thin–fat phenotype [70, 95]. However, in another study from India, inclusion of other variables resulted in two similar (SIDD and MARD) and two new (combined insulin-resistant and -deficient diabetes [CIRDD] and insulin-resistant obese diabetes [IROD]) clusters [96]. Integrating a PGS for insulin secretion or resistance (without actual measurements) with an existing risk tool in over 22,000 British Pakistani and Bangladeshi individuals still identified a probable severe insulin-deficient diabetes (pSIDD) subgroup, underscoring the robustness of these associations [97]. While the clinical variable-based approach seems less robust in populations of diverse ancestry, this could be attributed to differences in distribution (e.g. body composition) and genetic factors limiting the utility of PGSs in this context. Overall, this highlights the need for further refinement of clusters and inclusion of other important clinical variables, and the need to take into consideration parental and early life effects and population-specific definitions [70].

### Combined polygenic and phenotype approach

Combining genetics with phenotypic information derived from OGTTs, body fat distribution and liver fat content [98] resulted in six clusters of prediabetes characterised by low or very low type 2 diabetes risk, beta cell failure, low risk + obesity, high risk + insulin resistance + fatty liver, and high risk + visceral fat + nephropathy. This subgrouping targets underlying pathogenic defects to refine the stratification, but extension to non-European populations is still needed.

## Future perspectives

The share of heritability of type 2 diabetes that can be explained by identified variants has increased from 25% in the first GWAS decade (2007–2016) to about 50% currently. However, this means that at least half of the heritability is unexplained. In addition, type 2 diabetes risk loci have been shown to have sex-specific and parent-of-origin effects, which need to be taken into consideration to improve prediction. Moreover, the effects of gene–gene and gene–environment interactions are largely unknown. Future research and more affordable whole-genome sequencing will likely expand the numbers of common and rare genetic loci as well as ancestry-specific signals associated with diabetes, with particular efforts needed to identify ancestry-specific signals before clinical implementation can be tested. Examining the distribution of PGSs in different populations may then, perhaps in combination with clinical risk factors, allow for identification of groups who are at high risk of developing different subtypes of ‘type 2 diabetes’ and, especially, comorbidities. This approach could be piloted in a clinical setting to enable targeted interventions and lifestyle advice to be provided, to reduce the risk of developing the disease. Most importantly, it is hoped that PGSs could be used to identify individuals at risk of more aggressive disease forms to receive stringent monitoring and treatment, including targeted initiation of medication to prevent the development and progression of comorbidities. However, before that is feasible, further understanding is needed of the molecular mechanisms, biological functions and clinical implications of these loci.

The rapidly expanding healthcare-associated biobanks and direct-to-consumer genetic testing will facilitate not only gathering of genotype-associated follow-up data for risk evaluation, but also population-based implementation of PGSs. As technological advances render low-coverage whole-genome sequencing (which can be used to calculate PGSs and identify monogenic variants) less expensive, PGS construction will become increasingly cost-effective for preventive health approaches, particularly when performed early in life. The potential for applicability of PGSs in clinics worldwide will increase with more complete profiles of genetic risk variants across diverse populations. At the same time, pipelines for constructing PGSs (and updating them based on new knowledge), as well as interpreting them to aid clinicians and patients, are needed, together with frameworks connecting them to medical records.

To date, the accuracy of PGSs for type 2 diabetes is limited by our current understanding of the genetic causes of the disease. At best, they may improve our understanding of the underlying biology of type 2 diabetes and lead to the development of more effective preventive and therapeutic strategies. The variant-based clustering approaches represent a forerunner in this field. However, even if the soft clustering approach allows same variants to be included in different clusters, we need ways of analysing each individual’s profiles for multiple clusters and how they interact in providing the individual’s phenotype and affect risk estimates for comorbidities. This is likely to need solutions based on AI.

Although current knowledge cannot support the use of PGSs for diagnostic purposes, that is, to prove that an individual has type 2 diabetes, they could be used for elimination purposes, that is, to raise awareness of a high likelihood that an individual has a different type of diabetes and provide an incentive for screening for monogenic diabetes.

## Conclusion

Although high-throughput genomic studies have revealed significant breakthroughs in type 2 diabetes genetics and much of the heritability can be explained, we still lack insight into the molecular mechanisms, biological functions and clinical implications of most loci. Future research is also needed regarding population-specific genetics and missing heritability. Of note, despite the huge advances on the research side, translational pilot studies are scarce. The first use cases for PGSs in the clinic are likely to involve integrating them with current practice to predict high risk of diabetes or its comorbidities, which may lead to the development of pilot interventions for those at risk. The prediction of low risk of diabetes, without subsequent intervention, might be more cost-effective than targeting high-risk groups (given the presumably larger numbers of individuals affected), but this will be hard to accomplish given the current gaps in our knowledge.

## Supplementary Information

Below is the link to the electronic supplementary material.

Slideset of figures (PPTX 791 KB)

## Abbreviations

AI: Artificial intelligence
CAD: Coronary artery disease
GWAS: Genome-wide association study
MARD: Mild age-related diabetes
PGS: Polygenic score
pPS: Partitioned polygenic score
PXS: Polyexposure score
SIDD: Severe insulin-deficient diabetes
SIRD: Severe insulin-resistant diabetes
T2D-PGS: Type 2 diabetes polygenic score
