# Supplementary material for: Clinical use of polygenic scores in type 2 diabetes: challenges and possibilities
Source: Diabetologia. 2025 Apr 5;68(7):1361–74. doi: 10.1007/s00125-025-06419-1 (PMC12177005; doi:10.1007/s00125-025-06419-1)
Supplement: Supplementary file 1 — Slideset of figures (PPTX 791 KB) [file 125_2025_6419_MOESM1_ESM.pptx]

## Slide 1
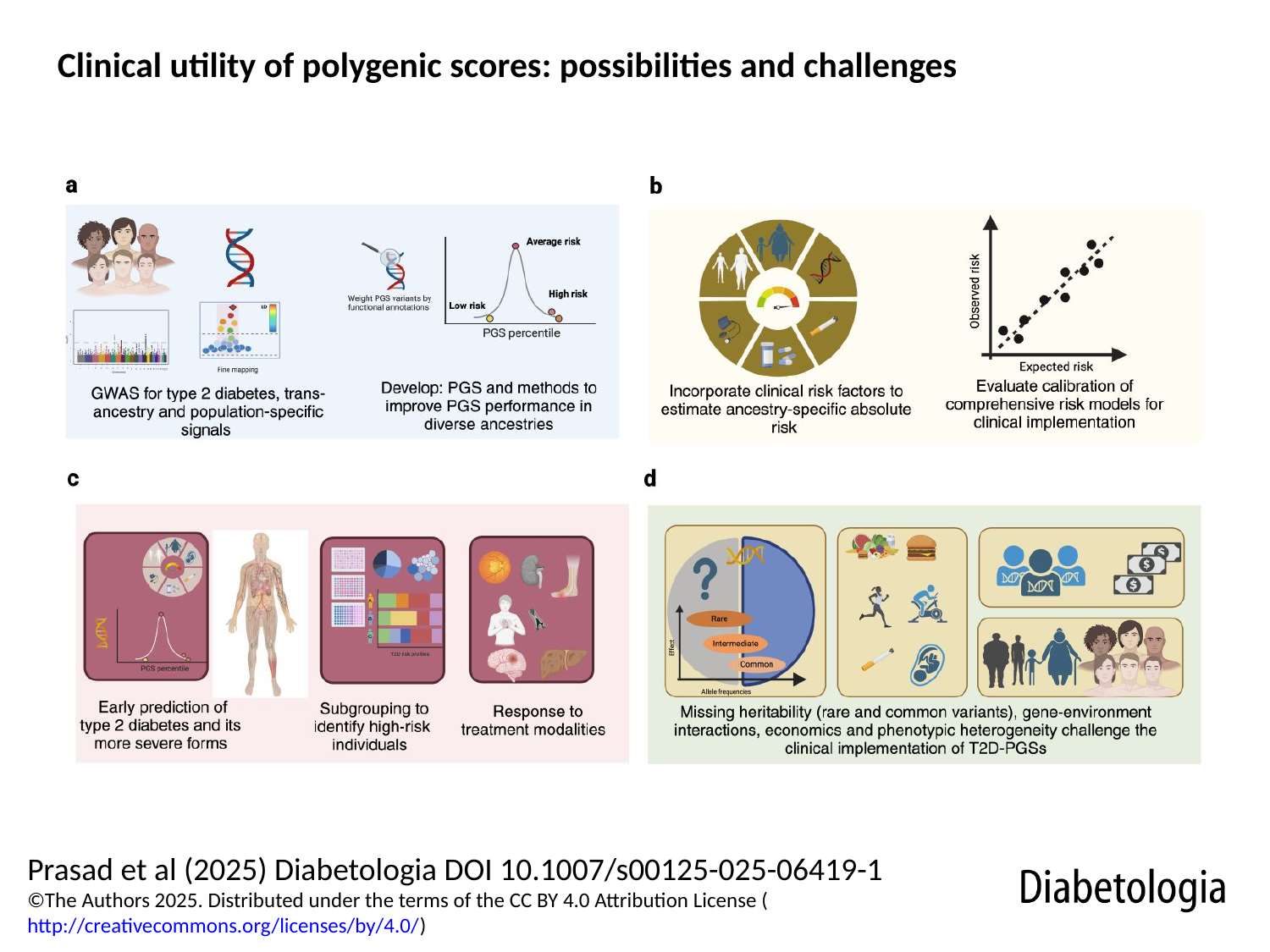

Clinical utility of polygenic scores: possibilities and challenges
Prasad et al (2025) Diabetologia DOI 10.1007/s00125-025-06419-1
©The Authors 2025. Distributed under the terms of the CC BY 4.0 Attribution License (http://creativecommons.org/licenses/by/4.0/)

## Slide 2
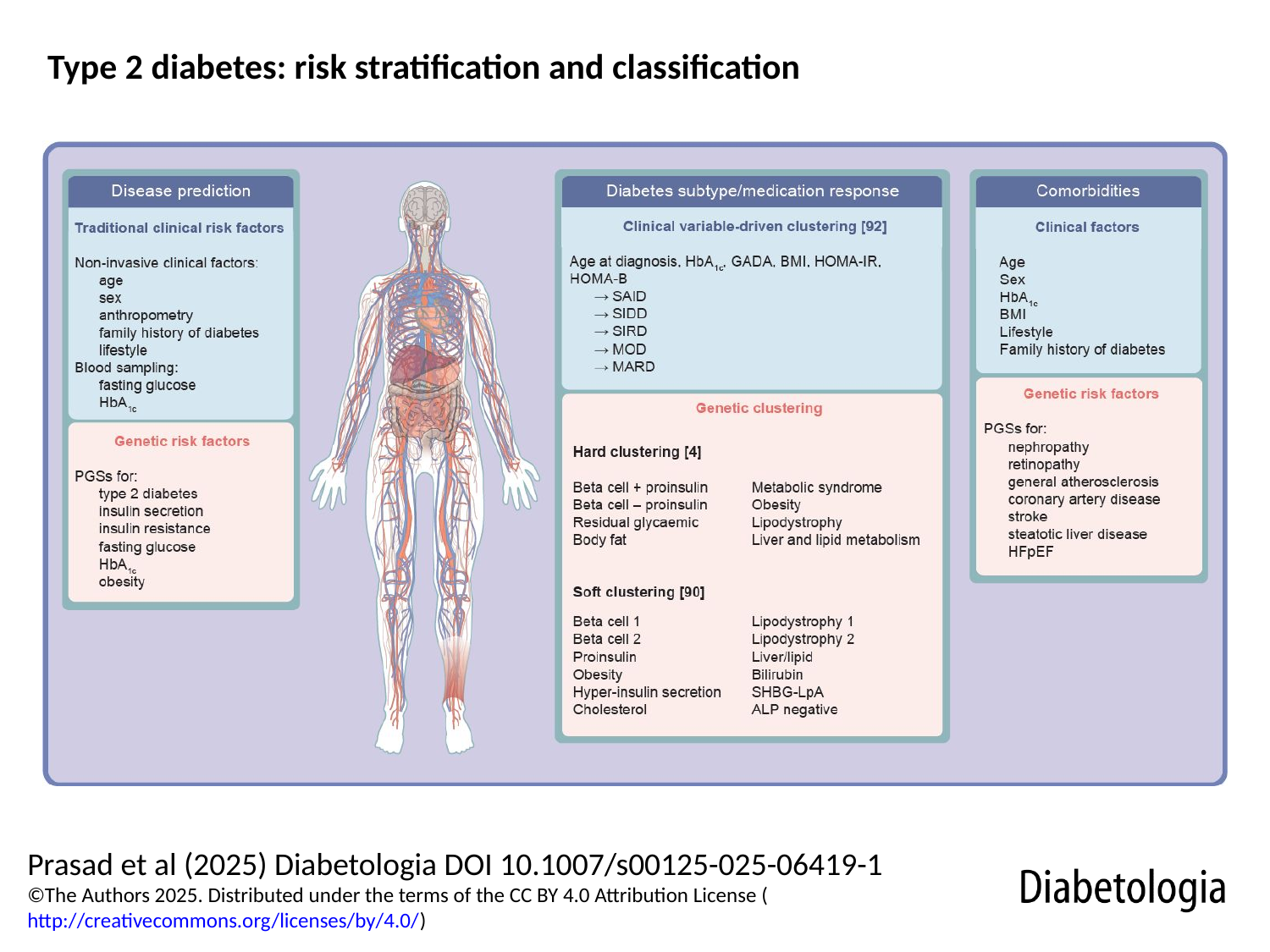

Type 2 diabetes: risk stratification and classification
Prasad et al (2025) Diabetologia DOI 10.1007/s00125-025-06419-1
©The Authors 2025. Distributed under the terms of the CC BY 4.0 Attribution License (http://creativecommons.org/licenses/by/4.0/)

## Slide 3
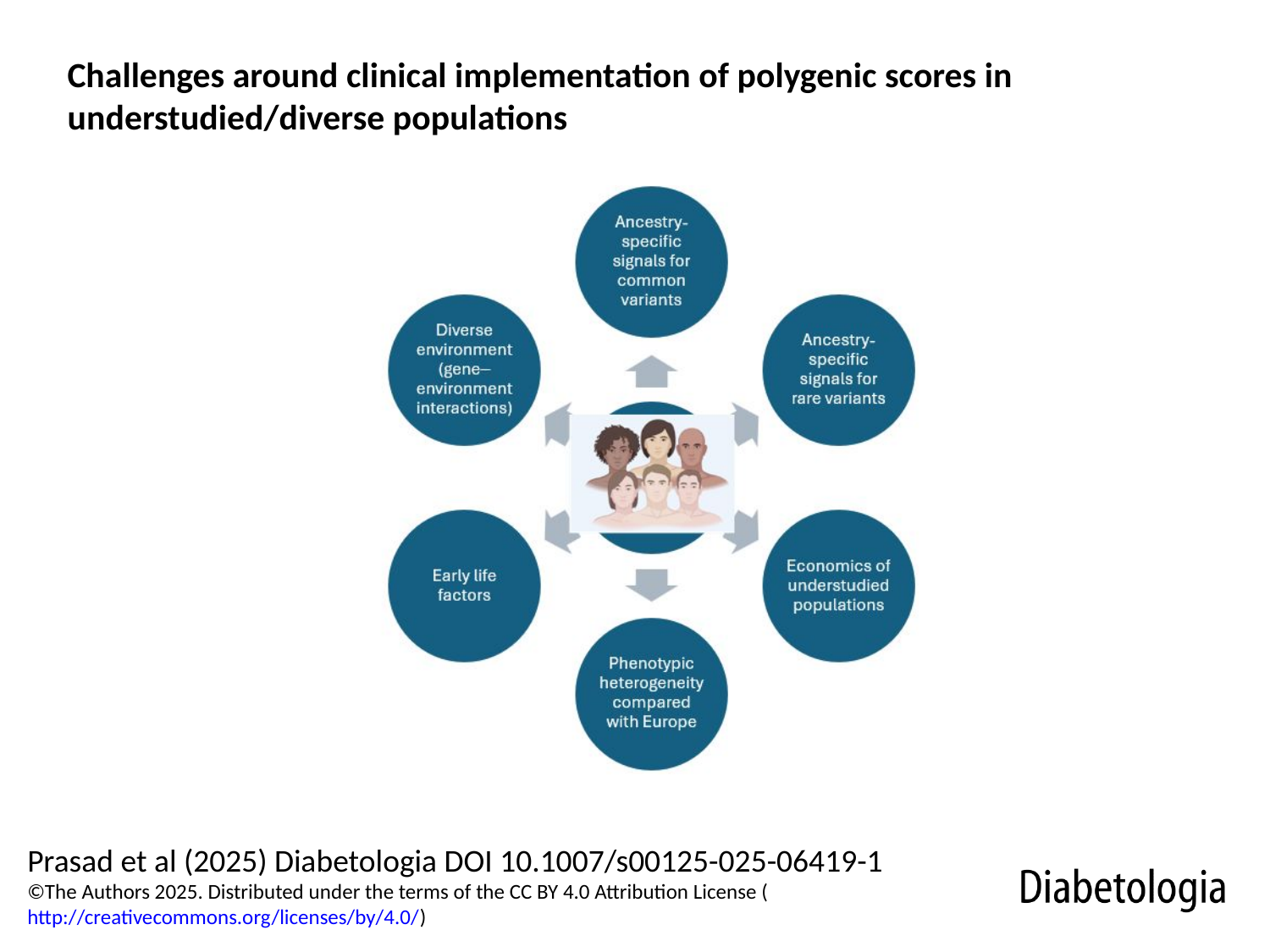

Challenges around clinical implementation of polygenic scores in understudied/diverse populations
Prasad et al (2025) Diabetologia DOI 10.1007/s00125-025-06419-1
©The Authors 2025. Distributed under the terms of the CC BY 4.0 Attribution License (http://creativecommons.org/licenses/by/4.0/)
